# The In-Vitro Effects of Sea Cucumber (Stichopus sp1) Extract on Human Osteoblast Cell Line

**DOI:** 10.5704/MOJ.1303.015

**Published:** 2013-03

**Authors:** A Shahrulazua, AR Samsudin, MA Iskandar, AS Amran

**Affiliations:** Department of Orthopaedics and Traumatology, Universiti Kebangsaan Malaysia Medical Centre, Kuala Lumpur, Malaysia; College of Dentistry, University of Sharjah, Sharjah, United Arab Emirates; Pantai Hospital Kuala Lumpur, Kuala Lumpur, Malaysia; Department of Orthopaedics, Universiti Sains Malaysia, Kubang Kerian, Malaysia

## Abstract

Despite its claimed therapeutic effects, the action of sea cucumber (known as gamat in the Malay language) on human osteoblast cells is still unknown. We performed in vitro studies utilising extract of Stichopus sp1 (gamat) to elucidate its effects on cell viability and functional activity. We found an inverse relationship between gamat concentration and its effect on osteoblast cell viability (p<0.001). Only gamat concentration at 1mg/ml significantly promoted cell viability at day 3 of incubation. There was a trend towards increased osteoblast cell function in the presence of gamat at 5mg/ml and 10mg/ml but this observation was not consistent at different incubation periods.

**Key Words:**

Alkaline phosphatase, Stichopus, Sea Cucumbers, Osteoblasts, Tetrazolium Salts

## Introduction

Sea cucumbers, also known as holothurians, are marine invertebrates living in shallow seawater, on reef flats and slopes with considerable hydrodynamic energy^1^. There are many holothuroidea species and Stichopus, one such example, is widely distributed across the tropical Indo- Pacific region 1. “Gamat”, Malay terminology for sea cucumber, is widely used orally and topically to treat various illnesses including low back pain and rheumatism^2^,^3^. Its usage is growing in popularity due to commercialisation, particularly in Malaysia^4^.

Various studies have shown that sea cucumber extract possesses several therapeutic properties such as a promoter of soft tissue healing^5^-^7^ and as an antibacterial^8^-^10^, antifungal^11^,^12^, antitumour^13^-^15^, antianaphylactic^16^, antiinflammatory^17^,^18^, antinociceptive 19-21 and antioxidant 22,23 agent. A previous study evaluating the effect of tibial bone fracture healing after oral administration of Stichopus sp1 extract in rabbit models showed improved fracture healing in the rabbits given a low dose (1mg/kg) of the extract^24^, but it remains to be elucidated whether this was due to a direct effect of the extract on bone cells or indirectly through some systemic mechanisms affecting bone metabolism. Thus, the objectives of this study were to determine the viability and functional activity of human osteoblast cells when grown in culture media supplemented with Stichopus sp1 extract at varying concentrations. Although there exist previous studies regarding the effect of sea cucumber extract on other cell lines such as fibroblast, osteoclast and endothelial cells 2, to date there are no studies in the literature about the effects of sea cucumber extract on human osteoblast cells. Osteoblasts play critical roles in the formation and mineralisation of bone matrix.

## Materials and Methods

This two-part laboratory study was approved by the ethics committee of ‘Universiti Sains Malaysia’. The aim of first phase was to elucidate optimal gamat concentrations. In the second part, we investigated whether the effect of gamat extract on osteoblast cells varied after different incubation periods. The outcomes of the study were measured by using MTT {(3-[4,5-dimethylthiazol-2-yl])-2,5-diphenyl tetrazolium bromide} assay following the methods described by Di Silvio 25 and recorded by an ELISA (Enzyme-linked immunosorbent assay) reader (Sunrise, Tecan, Austria) with the absorbance wavelength set at 570nm to investigate the cell viability^25^-^27^. Following the methods recommended by Di Silvio, we indirectly determined the cell functional activity using an ALP (alkaline phosphatase) assay to measure the concentration of p-nitrophenol at an absorbance wavelength of 405nm^28^-^30^. The negative control used was a standard culture media for osteoblast cell growth (a mixture of Dulbecco Modified Eagle’s Medium (DMEM) (Gibco, USA), 10% Fetal Bovine Serum (FBS) (BioWhittaker, USA) and 1% Penicillin / Streptomycin (Gibco, USA)^26^ . The positive control was 50% ethanol solution (HmbG Chemicals, Germany)^31^

We obtained a patented, purified water based extract of Stichopus Sp1 in powder form from an established local pharmaceutical company. This purified powder extract was sterilised using gamma radiation at 25 kGγ^32^. The sterilised powder extract was dissolved in standard culture media to various concentrations (1mg/ml, 5mg/ml, 10mg/ml, 20mg/ml, and 100mg/ml) and incubated at 370C temperature in a humidified atmosphere containing 5% CO₂ in air (ShelLab Model IR2424 CO₂ Incubator, USA) for 24 hours. The mixture was then filtered using 0.2μm pore size membranes (Sartorius, Germany) to remove excess powder particles. These test substances were added to wells of a 96- microplate (Nunc, Denmark) for use in both parts of the study.

First, a series of two fold dilutions of gamat extract from 100mg/ml down to 1.56mg/ml were prepared in 96 microplate wells. To each well, we added 10,000 osteoblasts cells (CRL-11372, ATCC, USA) so that for every gamat concentration tested, there were 12 wells prepared with 200μl solution. Similar number of wells containing negative control with the osteoblast cells was prepared. Twelve wells of positive control were prepared by mixing 100μl of 100% ethanol with 100μl of 10,000 osteoblast cells suspension in each well, making the final concentration of the ethanol solution equivalent to 50%. The prepared microplates were incubated for 72 hours at 370C with 5% CO₂, after which they were tested quantitatively using MTT and ALP assays. Results were also recorded qualitatively using an Axiovert 40C Inverted Microscope (Carl Zeiss, Germany). Each experiment was repeated twice.

We chose four gamat concentrations (1mg/ml, 5 mg/ml, 10mg/ml, and 20mg/ml) for the second phase of the study, based on results of the first study and the calculated cytotoxic dose (IC₅₀), which is the concentration of the test substance that reduces the number of cells by 50% as compared to untreated cells. For this purpose, 10,000 osteoblast cells in 100μl of complete media were first seeded in each well of a 96 well microplate and incubated at 37°C with 5% CO₂. After 24 hours, the media in the wells was removed, leaving just the cells. Each well containing the cells was then mixed with 100μl extract of the four chosen gamat concentrations and a separate negative control so that for each test substance, there were 9 wells prepared. The plates were then incubated at 37°C with 5% CO₂ until MTT and ALP assays were performed after one hour, one day, 3 days, 5 days and 7 days of incubation period. All tests were done in triplicates.

A simple chemical analysis (Na⁺, K+, Cl−, glucose, total protein, triglyceride and cholesterol) was also performed on the gamat extract dissolved in distilled water at 100mg/ml and 20mg/ml concentrations after 24 hours of incubation (5% CO₂, 37°C) using a Hitachi 912 Automatic Analyser (Boehringer Mannheim, Germany). Osmolarity of the various gamat-culture media solutions used in this study (1mg/ml, 5mg/ml, 10mg/ml, 20mg/ml, and 100mg/ml) including the negative control was measured using an Osmomat 030-D Cryoscopic Osmometer (Gonotec, Germany) and the pH readings of these different solutions were tested using a pH 211 Microprocessor pH Metre (Hanna instruments, USA). The mean values of 3 different sets of measurements were recorded for the chemical analysis, osmolarity, and pH tests.

We used SPSS software, version 12.0.1 for Windows (SPSS Inc., USA) for statistical analyses. All readings for each concentration of the gamat tested and the controls were analysed by combining the results from the 3 separate experimental runs. Results were evaluated by analysis of variance (ANOVA) and post hoc Bonferroni tests. Significance level was set at p<0.05 for all statistical tests. Data were presented as means with 95% confidence interval (CI).

## Results

**Fig. 1:**
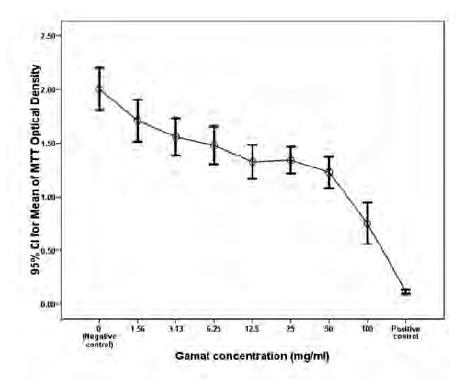
: Osteoblast cell viability was significantly decreased for all tested gamat concentrations (1.6mg/ml, 3.1mg/ml, 6.3mg/ml, 12.5mg/ml, 25mg/ml, 50mg/ml, and 100mg/ml) when compared to the negative control after 3 days of incubation (p<0.001).

Results of simple chemical analysis, osmolarity and pH tests for the various solutions are shown in Table I and II. Results of the first phase showed that cell viability was significantly decreased for all tested gamat concentrations (1.6mg/ml, 3.1mg/ml, 6.3mg/ml, 12.5mg/ml, 25mg/ml, 50mg/ml, and 100mg/ml) when compared to the negative control after 3 days of incubation (p<0.001) (Figure 1).

**Fig. 2:**
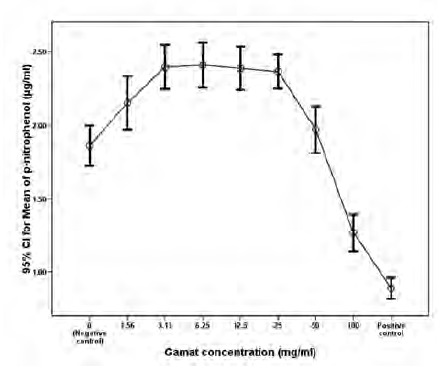
: P-nitrophenol measurements were significantly higher for each gamat concentration at 1.6mg/ml, 3.1mg/ml, 6.3mg/ml, 12.5mg/ml, and 25mg/ml as compared to the negative control after 3 days of incubation (p=0.016 for 1.6mg/ml; p<0.001 for the other 4 concentrations).

The IC₅₀ was estimated at 75mg/ml. In contrast, the ALP assay showed that p-nitrophenol measurements were significantly higher for each gamat concentration at 1.6mg/ml, 3.1mg/ml, 6.3mg/ml, 12.5mg/ml, and 25mg/ml as compared to the negative control after 3 days of incubation (p=0.016 for 1.6mg/ml; p<0.001 for the other 4 concentrations) (Figure 2). Among these 5 gamat concentrations, there was no significant statistical difference in osteoblast cell functional activity. There was also no significant statistical difference in the effects of osteoblast cell activity when gamat concentration of 50mg/ml was compared with the negative control and 1.6mg/ml concentration. There was a statistically significant reduction in cell activity with gamat concentrations of 50mg/ml when compared individually to 3.1mg/ml, 6.3mg/ml, 12.5mg/ml and 25mg/ml concentrations.

**Fig. 3:**
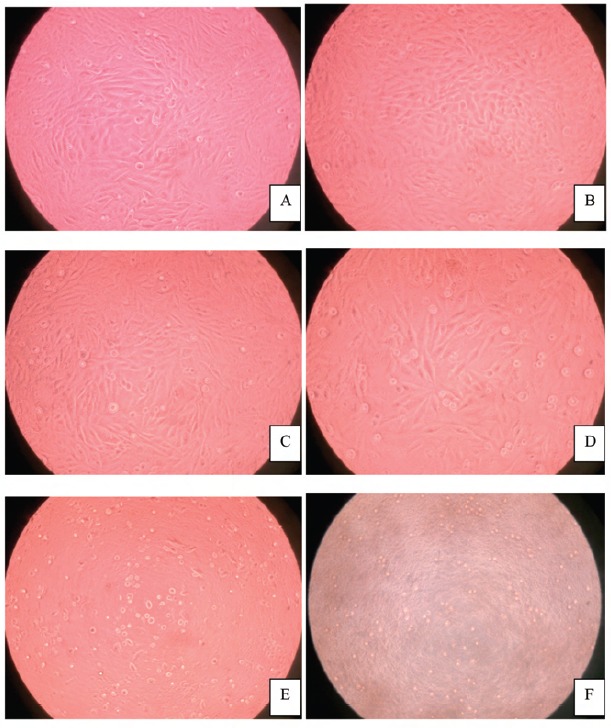
: Microscopic appearance of osteoblast cells after three days of incubation period, viewed under x20 magnification, in their corresponding growth media (A, negative control;B, gamat at 1.6mg/ml; C,gamat at 25mg/ml; D, gamat at 50mg/ml; E, gamat at 100mg/ml; F=positive control).

Microscopic observations (Figure 3) demonstrated an unhealthy cell lineage in wells containing high concentration of gamat extracts. Confluency of the wells was similar in the negative control and in wells containing extract of gamat concentration from 1.6mg/ml up to 25mg/ml; confluency was reduced in the wells containing 50mg/ml and 100mg/ml of gamat extract. In the wells containing gamat concentration of 100mg/ml, the osteoblast cells became smaller and grainy in appearance similar to those of the positive controls.

**Fig. 4:**
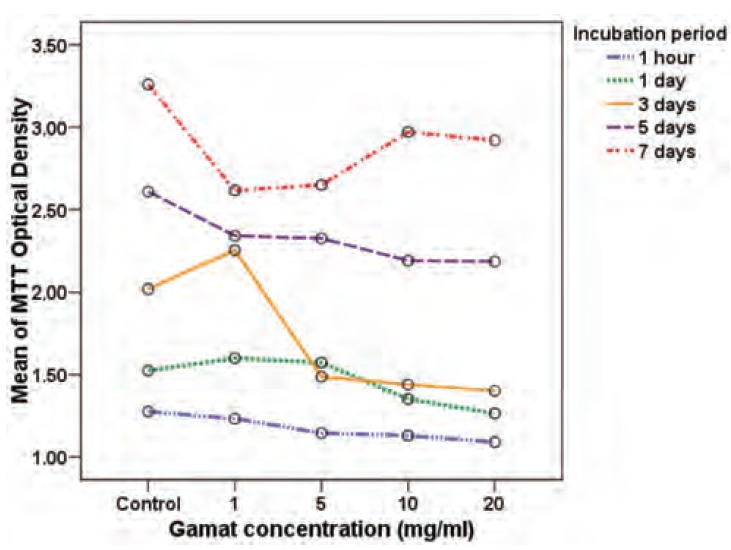
: Effect on osteoblast cells viability by various gamat concentrations after different incubation periods.

**Fig. 5:**
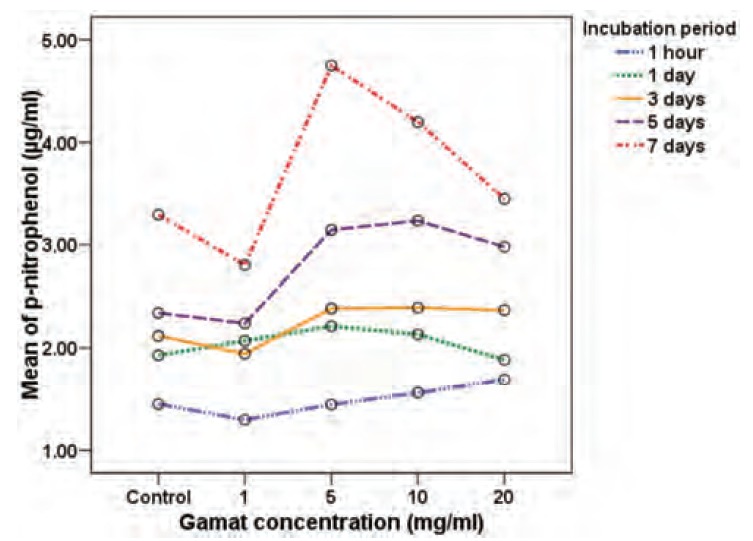
: Effect on osteoblast functional activity by various gamat concentrations after different incubation periods.

Multi-factorial ANOVA analysis showed that, in general, after adjusting for the effect of duration of incubation and different experimental runs, there was a significant difference in the osteoblast cell viability and functional activity in different gamat concentrations (p<0.001). Importantly, there was a significant interaction between the concentration effect and the duration of incubation (p<0.001). In figures 4 and 5, we show that the effect of concentration was not parallel with the effect of various incubation durations. In general, osteoblast cell viability and functional activity increased as incubation time increased, but the effect of each gamat concentration varied with different incubation periods when compared to the negative control in terms of osteoblast cell viability and activity. We therefore conducted individual comparisons of the concentration effect for each incubation period, and these results are presented in Table III and IV. Table III shows that at most incubation times, there was a trend towards decreasing osteoblast cell viability with increased gamat concentration when compared to the negative control. There was however a significant increase in osteoblast viability at Day 3 of incubation with gamat at 1mg/ml when compared to the negative control (p=0.011). Table IV highlights a significantly greater promoting effect on alkaline phosphatase expression with gamat at 5mg/ml and 10mg/ml compared to the negative control, for Day 1, Day 5 and Day 7 of incubation.

## Discussion

Although the results of the first part of this study showed an inverse relationship between gamat concentration and osteoblast cell viability, there was a positive promoting effect of gamat extract on osteoblast functional activity when 1.6mg/ml, 3.1mg/ml, 6.3mg/ml, 12.5mg/ml, and 25mg/ml of gamat concentrations were used. Microscopic examination showed adequate cell confluency in the wells with gamat concentration from 1.6mg/ml up to 25mg/ml. Taking these observations and the estimated IC₅₀ into account, gamat concentration of 1mg/ml, 5 mg/ml, 10mg/ml, and 20mg/ml were chosen for the second part of the study in an attempt to investigate the effects of gamat on osteoblast viability and functional activity after different incubation periods. This was done because the rate of in-vitro cell growth and activity would vary at different incubation times and hence would influence the results and final conclusions of this study.

Apart from cell apoptosis, a decrease in cell viability should theoretically be accompanied by a reduction in cell activity. The fact that there was still a significant increment of osteoblast activity at gamat concentrations of 1.6mg/ml, 3.1mg/ml, 6.3mg/ml, 12.5mg/ml, and 25mg/ml when compared to the negative control, despite a decrease in cell viability was an interesting finding. It is possible that after the treatment with these gamat concentrations, the cell metabolism was increased to such an extent that the metabolites by-products released by the cells caused toxicity in the culture system, leading to increased cell death. However, this would not explain why at 50mg/ml concentration, there was still a significantly decreased in cell viability despite a relatively insignificant change in cell functional activity.

Next, we demonstrated that, as expected, there were more viable osteoblast cells with prolonged incubation due to the increased time allowed for inherent cell division and proliferation. At incubation Day 3, there was a significant increase in cell viability with gamat at 1mg/ml when compared to the negative control. This observation was not seen at the other four incubation periods, hence it could represent an outlier and its significance was questionable. Moreover, there was no parallel increase in the cell activity with this gamat concentration at the incubation Day 3.

As expected, the findings noted in the second part of the study were rather different than those observed in the first study at incubation Day 3. The difference is possibly explained in relation to the actual technique used in the preparation of the osteoblast cells in the wells of the microplates. In the first phase, cells were mixed with culture media containing the gamat extract before they were incubated for 3 days, whereas in the latter, cells were primarily seeded in the wells for 24 hours to allow for cell attachment before they were mixed with the test substances. Additionally, the osteoblast cell lines used in the first and second part of the study were derived from different cell passages, which might influence on the cell differentiation capability.

In accordance with the results of an in-vivo study that demonstrated an overall delay and possibly cytotoxic effect of high dose oral gamat on fracture healing^24^, we found that gamat extract reduces osteoblast cell viability in a concentration-dependent manner compared to the control media. Interestingly, other studies have shown that the extract of Stichopus species also affects viability or proliferation of human fibroblasts and osteoclast cells in a negative manner^31^,^33^. In the present study, we did not find a consistent positive effect of the Stichopus extract on human osteoblast cell viability, even at the lowest concentrations (1mg/ml) tested. In contrast, Shaifuzain et al. reported a more favourable fracture healing response in rabbit models given low doses (1mg/kg) of oral gamat^24^. The improved fracture healing response seen in their study may be due to a systemic effect of the gamat rather than a direct effect on osteoblast cells. The increased functional activity of the osteoblast cells that we observed with gamat at 5mg/ml and 10mg/ml at certain incubation periods suggests that gamat extract may possibly act as a morphogen, causing increased osteoblast cell expression and differentiation, even if it has no direct role as a mitogen.

Previous studies showed that the water extract of Stichopus contains high amino acid concentrations (37%)34 as well as calcium, magnesium, iron and zinc^2^ that may play an important role in osteoblast molecular activities. Although the effect of glycosaminoglycan (GAG) such as chondroitin sulphate, commonly found in sea cucumber extract^2^,^35^, was previously shown to inhibit osteoblast cell proliferation in vitro36, oral administration of chondroitin sulphates had been shown to increase the total calcium pool and intestinal absorption of calcium^37^, which may lead to an increased capacity for injured bone to regenerate during osteogenesis. The exact mechanism by which gamat affects osteoblasts and human bone however remains unclear and is outside the scope of this study. Isolation of the bioactive compounds was not performed in this study since the aim was to investigate the effect of the basic extract of local gamat, which is often the one used by patients and readily available in the market.

Previous studies have shown that pH and osmolarity affect cell growth in vitro^31^,^38^. We confirmed that there was no marked difference in the pH values and osmolarity among the various gamat concentrations as well as the negative control that could account for any discrepancy noted in the osteoblast proliferation and viability. Our chemical analysis confirmed that this gamat extract did not contain high concentrations of sodium and chloride salts, which are naturally present in high levels in seawater from which the Stichopus species are harvested. High levels of these salts in the extract could lead to high osmolarity cytotoxic solutions^31^. Anderson et al. found that the endogenous hormones present in foetal bovine serum could potentially mask the effect of any exogenous growth factor added to a culture system^39^, but in reality, human osteoblast cells do not exist in a “serum-free” environment and in fact, are exposed to many plasma proteins comparable to those found in foetal bovine serum. Hence, the results of this study are more likely to approximate the clinical environment by mixing the gamat extract with a culture media containing serum. Furthermore, the presence of serum possibly buffers the cell culture system against various disturbances and toxic effects such as pH change, presence of heavy metals, proteolytic activity, and endotoxin^40^. Nonetheless, a repeat of this study should be conducted using serum-free media in order to completely eliminate the possibility of interaction between substances in the serum and any exogenous growth factors that might be present in gamat extract.

For future research, a similar study with smaller number of cells per well should be conducted in order to allow more room for cell growth. Likewise, a lower concentration of gamat (<1mg/ml) should be tested. Further, experiments should be conducted with shorter incubation periods, for example at 4-hourly intervals during the first 24 hours. Those findings would answer the question of whether a burst of overgrowth or over-activity causes early saturation and overcrowding in the cell population, subsequently causing a decrease in further cell growth. Another inherent limitation with the current study is that it is difficult to ascertain whether the observed reduction in MTT measurements was due to reduced cell proliferation or to toxicity stemming from the gamat extract. Further studies to identify the active component(s) of the extract, elucidate its structure and verify its toxicity properties on osteoblasts are therefore recommended.

**Table I:**
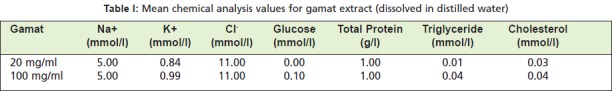
: Mean chemical analysis values for gamat extract (dissolved in distilled water)

**Table II:**
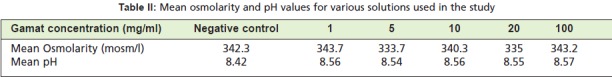
: Mean osmolarity and pH values for various solutions used in the study

**Table III:**
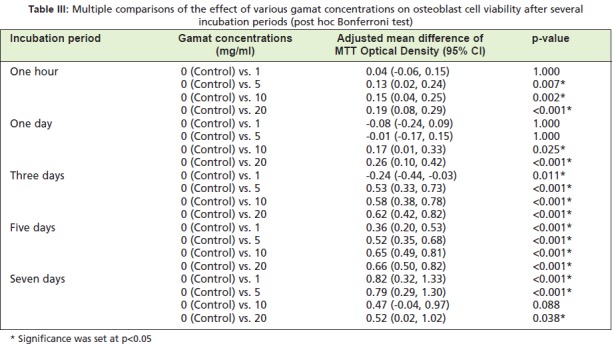
: Multiple comparisons of the effect of various gamat concentrations on osteoblast cell viability after several incubation periods (post hoc Bonferroni test)

**Table IV:**
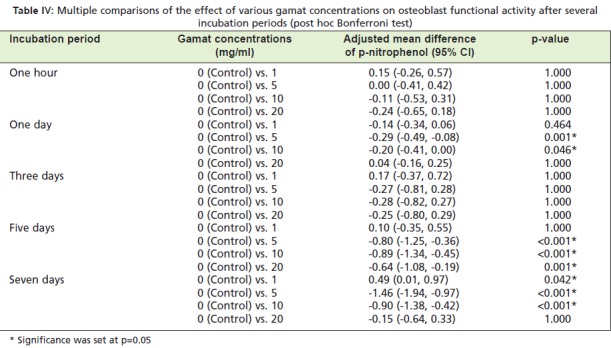
: Multiple comparisons of the effect of various gamat concentrations on osteoblast functional activity after several incubation periods (post hoc Bonferroni test)

## Conclusion

Although sea cucumber extract of Stichopus Sp1 appears to reduce human osteoblast cell viability in a concentrationdependent manner, it may potentially promote osteoblast functional activity. Further research is therefore essential to investigate the possible role of sea cucumber extract as a systemic modulator of human bone metabolism.
